# Preferred Imaging for Target Volume Delineation for Radiotherapy of Recurrent Glioblastoma: A Literature Review of the Available Evidence

**DOI:** 10.3390/jpm14050538

**Published:** 2024-05-17

**Authors:** Francesco Cuccia, Fatemeh Jafari, Salvatore D’Alessandro, Giuseppe Carruba, Giuseppe Craparo, Giovanni Tringali, Livio Blasi, Giuseppe Ferrera

**Affiliations:** 1 Radiation Oncology, ARNAS Civico Hospital, 90100 Palermo, Italygiuseppe.ferrera@arnascivico.it (G.F.); 2 Radiation Oncology Department, Imam-Khomeini Hospital Complex, Teheran University of Medical Sciences, Teheran 1416634793, Iran; 3 Division of Internationalization and Health Research (SIRS), ARNAS Civico Hospital, 90100 Palermo, Italy; 4 Neuroradiology, ARNAS Civico Hospital, 90100 Palermo, Italy; 5 Neurosurgery, ARNAS Civico Hospital, 90100 Palermo, Italy; giovanni.tringali@arnascivico.it; 6 Medical Oncology, ARNAS Civico Hospital, 90100 Palermo, Italy; livio.blasi@arnascivico.it

**Keywords:** re-irradiation, glioblastoma, radiotherapy, imaging

## Abstract

Background: Recurrence in glioblastoma lacks a standardized treatment, prompting an exploration of re-irradiation’s efficacy. Methods: A comprehensive systematic review from January 2005 to May 2023 assessed the role of MRI sequences in recurrent glioblastoma re-irradiation. The search criteria, employing MeSH terms, targeted English-language, peer-reviewed articles. The inclusion criteria comprised both retrospective and prospective studies, excluding certain types and populations for specificity. The PICO methodology guided data extraction, and the statistical analysis employed Chi-squared tests via MedCalc v22.009. Results: Out of the 355 identified studies, 81 met the criteria, involving 3280 patients across 65 retrospective and 16 prospective studies. The key findings indicate diverse treatment modalities, with linac-based photons predominating. The median age at re-irradiation was 54 years, and the median time interval between radiation courses was 15.5 months. Contrast-enhanced T1-weighted sequences were favored for target delineation, with PET-imaging used in fewer studies. Re-irradiation was generally well tolerated (median G3 adverse events: 3.5%). The clinical outcomes varied, with a median 1-year local control rate of 61% and a median overall survival of 11 months. No significant differences were noted in the G3 toxicity and clinical outcomes based on the MRI sequence preference or PET-based delineation. Conclusions: In the setting of recurrent glioblastoma, contrast-enhanced T1-weighted sequences were preferred for target delineation, allowing clinicians to deliver a safe and effective therapeutic option; amino acid PET imaging may represent a useful device to discriminate radionecrosis from recurrent disease. Future investigations, including the ongoing GLIAA, NOA-10, ARO 2013/1 trial, will aim to refine approaches and standardize methodologies for improved outcomes in recurrent glioblastoma re-irradiation.

## 1. Introduction

Glioblastoma (GBM) is the most prevalent form of brain malignancy in adults, posing a significant challenge in the field of oncology. The conventional method for addressing this highly aggressive tumor involves an initial extensive surgical resection, with the goal of maximizing the removal of cancerous tissue, followed by subsequent adjuvant chemoradiotherapy. Despite the application of these standard therapeutic approaches, the prognosis for individuals suffering from GBM remains disheartening, primarily due to the widespread occurrence of local failure. The persistent nature of GBM highlights the urgent need for innovative strategies and therapeutic breakthroughs to substantially improve the overall outcomes and enhance the quality of life for affected individuals [1].

Moreover, recurrent disease represents a challenging scenario from a clinical perspective, due to the heterogeneity of disease presentation, both from a radiological and patient-related point of view. Thus, due to the lack of standardized criteria, the best treatment option is often personalized, based on the multidisciplinary assessment of the performance status, disease presentation (small volume vs. infiltrative growth), previous treatments and expected impact on the natural history of the disease.

The ongoing discourse surrounding target volume delineation for post-operative glioblastoma radiotherapy, shaped by the tumor’s distinctive infiltrative growth, remains intricate. Despite evidence indicating that larger treatment volumes do not improve survival and may elevate the risk of neurotoxicity, the identification of optimal targets poses a persistent challenge. The recently updated ESTRO-EANO guidelines offer vital insights, stemming from an extensive investigation led by the ESTRO Guidelines Committee and European experts. Employing a meticulous two-step Delphi process, they addressed key aspects, including pre-treatment measures, immobilization, target delineation using both standard and innovative imaging, and technical considerations such as planning techniques and fractionation. The guidelines propose a unified clinical target volume definition based on postoperative contrast-enhanced T1 abnormalities, recommending a reduced 15 mm margin in accordance with the EORTC recommendation. Special scenarios are acknowledged, allowing for potential adjustments based on specific clinical contexts. Emphasizing alignment with the EORTC consensus, the guidelines endorse isotropic margins without cone-down procedures. Underscoring the importance of personalized planning target volume (PTV) margins, particularly in the context of image-guided radiation therapy (IGRT), the guidelines suggest a margin typically not exceeding 3 mm. This comprehensive guidance adeptly navigates the intricacies of target volume delineation, significantly contributing to ongoing initiatives aimed at refining post-operative radiotherapy for glioblastoma patients while minimizing the associated risk of neurotoxicity [2].

However, the striking frequency of local relapse in up to 90% of patients underscores the pressing need for effective interventions, given the absence of a current standard treatment. While the surgical approach remains a viable choice when feasible, it brings with it the potential for post-operative complications, necessitating a careful balance between therapeutic benefits and the associated risks. Brachytherapy, serving as an alternative, provides a localized therapeutic strategy for managing GBM recurrence. Nevertheless, it poses challenges, particularly an elevated risk of infection and/or hemorrhage, prompting a thorough consideration of the potential benefits against possible adverse outcomes. In addition to these alternatives, ongoing research delves into innovative methods like immunotherapy and targeted molecular therapies, aiming to amplify treatment effectiveness and prolong disease-free intervals. The intricacies of GBM demand a comprehensive approach, underscoring the importance of personalized treatment plans tailored to individual patient profiles in the continual pursuit of improving outcomes for those confronting GBM recurrence [3].

External beam radiotherapy (EBRT) stands as a commonly chosen treatment method, often selected with conventional fractionation or hypofractionated/stereotactic radiotherapy for glioblastoma multiforme management. The fractionation schedule’s importance persists, favoring smaller volumes in re-irradiation scenarios, guided by gadolinium-enhanced T1-weighted MR sequences for target volume delineation. While EBRT is established, recent investigations spotlight the potential of dose-escalated radiation therapy (DE-RT) for newly diagnosed glioblastoma multiforme patients. A systematic review and meta-analysis comparing overall survival (OS) and progression-free survival (PFS) between DE-RT and standard-of-care radiation therapy (SoC-RT) revealed that DE-RT alone outperforms SoC-RT alone in 1-year OS and demonstrates higher 1-year PFS. However, when combined with temozolomide (TMZ), DE-RT does not show superior outcomes over SoC-RT with TMZ. Interestingly, the study emphasizes the lack of a distinct benefit based on MGMT status, underscoring the necessity for future studies to precisely identify subgroups deriving the most significant benefit from DE-RT. This evolving evidence enhances our nuanced comprehension of glioblastoma treatment options, underscoring the dynamic nature of therapeutic approaches in this challenging clinical landscape [4].

However, some authors stress the importance of incorporating T2-weighted sequences into glioblastoma treatment plans to address edema during relapse, necessitating larger treatment volumes. This aligns with the evolving approach to glioblastoma management, utilizing advanced imaging for nuanced insights. Turning to treatments, the standard for newly diagnosed glioblastoma involves concurrent temozolomide-radiotherapy, followed by maintenance temozolomide. Bevacizumab, approved for recurrent cases, underwent scrutiny in a randomized trial with 978 patients. Although the differences in overall survival were insignificant, bevacizumab extended progression-free survival. Adverse events included modest increases in hypertension, thromboembolic events, intestinal perforation, and neutropenia. The trial highlighted evolving symptom burdens and neurocognitive function decline, revealing the complexities of therapeutic decisions for newly diagnosed glioblastoma [5].

In recent years, several authors have highlighted the potential utility of metabolic imaging in accurately assessing the extent of recurrent disease. Two studies provide valuable insights into this application. The first study, by Moreau et al., explores the feasibility of using ^68^Ga-prostate-specific membrane antigen (PSMA) 11 PET/CT in comparison with ^18^F-FDOPA PET/CT to detect early recurrence in glioblastoma patients. Despite some discrepancies, the study underscores the potential role of ^68^Ga-PSMA-11 PET/CT in discriminating between postradiation inflammation and recurrence, emphasizing the need for further prospective studies to validate these findings [6]. The second study, by Şahin et al., investigates the contribution of ^68^Ga-PSMA PET to defining the radiotherapy target volumes for glioblastoma, comparing the results with magnetic resonance imaging (MRI). The study reveals that ^68^Ga-PSMA PET can assist in delineating non-enhancing tumor parts and recurrent tumor volumes, suggesting its potential in reirradiation scenarios. These findings contribute to the evolving understanding of the role of ^68^Ga-PSMA PET in imaging glial tumors and its potential benefits in target volume delineation, especially in recurrent tumors [6,7].

Debates persist regarding the ideal imaging modality for glioblastoma re-irradiation, with c.e. T1-weighted sequences exposing more vascularized disease and T2-weighted or FLAIR imaging revealing microscopic disease, peritumoral edema with microscopic spread, and/or low-grade compartment of the gliomas, albeit with increased confounding features from prior chemo-radiotherapy. Findings from Constine et al.’s study shed light on the intricacies of brain irradiation effects. The study underscores MR imaging’s sensitivity, detecting abnormalities linked to larger treatment volumes, higher doses, or advanced age. Unlike CT, which revealed abnormalities in only 35% of patients, MR imaging identified an increased signal intensity in all cases. Higher-grade MR lesions were associated with larger volumes and higher doses, even in hyperfractionated schedules. Clinical abnormalities, such as impaired mental functioning and learning disabilities, correlated with MR-detected changes, underscoring the pivotal role of advanced imaging in comprehending the nuanced effects of brain irradiation [8].

Bell et al. recently explored the potential role of spectroscopic MRI (sMRI) in improving GBM re-irradiation within a prospective study involving 14 patients. Their investigation suggests the efficacy of spectroscopic imaging for implementing dose-escalated treatment plans. The 2023 study by Bell et al. revealed a correlation between GBM cellularity and the mapping of the whole-brain sMRI-generated relative choline to N-Acetyl-Aspartate ratio (rChoNAA). In the context of recurrent GBM (rGBM), where delineating the tumor volume (TV) poses challenges, the study demonstrated the usefulness of rChoNAA maps for precise re-RT targeting. Notably, the study found that rChoNAA  >  2 volumes were significantly larger, showcasing their potential use in optimizing treatment strategies. Ongoing research aims to validate the utility of sMRI in recurrent GBM, particularly in the context of dose escalation and understanding failure patterns [9].

Given the ongoing debate surrounding the selection of the most suitable imaging for target volume delineation, this systematic review endeavors to assess the favored imaging modalities for re-irradiation in GBM. The primary focus lies on the MR sequences utilized and the potential collaborative impact of metabolic examinations. The critical evaluation aims to provide insights into refining the approach to GBM re-irradiation, considering the complexities associated with optimal imaging choices. Understanding the interplay between MR sequences and metabolic exams is essential in enhancing the precision and effectiveness of re-irradiation strategies for glioblastoma.

## 2. Methods

In June 2023, a comprehensive systematic review of the literature spanning from January 2005 to May 2023 was conducted, focusing specifically on the role of MRI sequences in recurrent glioblastoma re-irradiation. The search criteria involved the meticulous usage of MeSH terms, including re-irradiation, repeated radiotherapy, stereotactic radiotherapy, radiosurgery, and recurrent glioblastoma. The articles considered for inclusion were limited to those in the English language and published in peer-reviewed journals. Both retrospective and prospective studies were encompassed, with a deliberate exclusion of brachytherapy experiences for the study’s specific objectives. Additionally, an exhaustive manual bibliography search was undertaken for all eligible articles. To maintain precision, certain manuscript types were excluded, such as case reports, reviews, planning studies, study protocols, and technology studies lacking clinical outcome data. Studies involving a mixed group of previously irradiated and unirradiated patients or pediatric patients were also omitted from the analysis. This systematic review aims to provide a comprehensive and insightful analysis of the existing literature, shedding light on the nuanced role of MRI sequences in the context of recurrent glioblastoma re-irradiation.

Two authors (FC and FJ) screened the articles that were potentially eligible for this study, with a third independent reviewer (GF) involved in the case of disagreement between the first two.

For the present analysis, a meticulous review of relevant publications was conducted, extracting essential features to comprehensively understand the landscape of recurrent glioblastoma re-irradiation. Key parameters, including the first author, publication year, study design, patient numbers, median age at re-irradiation, previous radiation therapy (RT) dose, time intervals between RT, IDH and MGMT status at recurrence, PTV volume, re-irradiation regimens, use of simultaneous integrated boost (SIB), preferred MRI sequences, utilization of PET for target volume delineation, concurrent systemic therapy, and clinical outcomes (local control, LC; progression-free survival, PFS; overall survival, OS) and toxicity, were systematically derived from each source. The literature review was meticulously executed following the Population, Intervention, Comparison, and Outcome (PICO) methodology, a robust approach commonly employed in evidence-based medicine to discern the components of clinical evidence for systematic reviews [10].

Baseline characteristics were systematically captured to generate descriptive statistics. A comprehensive comparison of the clinical outcomes was conducted through the Chi-squared test, with a significance threshold set at a *p*-value < 0.05. MedCalc v22.009, based in Marienkirche, Belgium, served as the statistical analysis tool for these evaluations.

## 3. Results

### 3.1. Population

**Figure 1:**
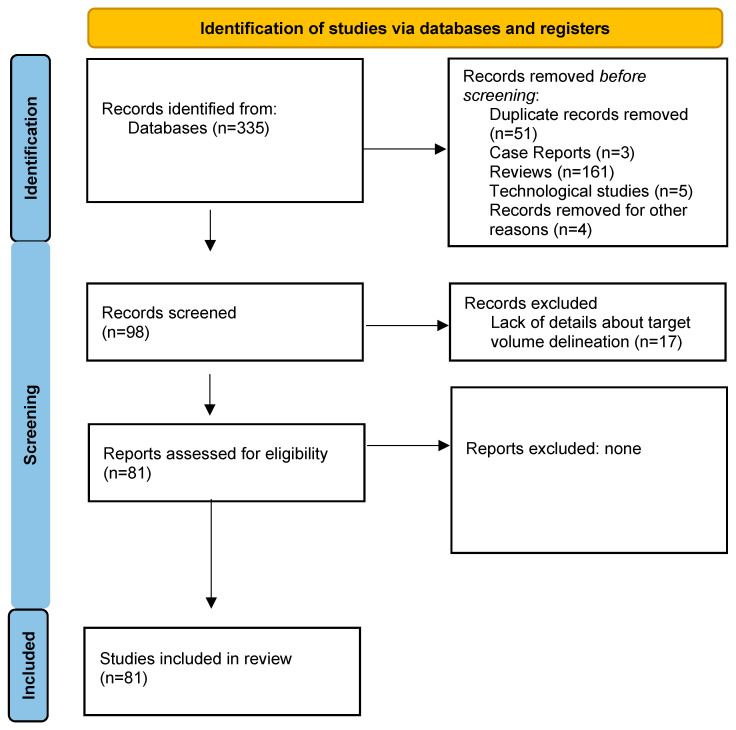
PRISMA flowchart. [91].

A total of 355 studies were identified for the purpose of this review, and 98 matched the search criteria. Of these, 17 were excluded due to their lack of details about target volume delineation; this resulted in a total of 81 studies, 65 retrospective and 16 prospective, enrolling 3280 patients [11,12,13,14,15,16,17,18,19,20,21,22,23,24,25,26,27,28,29,30,31,32,33,34,35,36,37,38,39,40,41,42,43,44,45,46,47,48,49,50,51,52,53,54,55,56,57,58,59,60,61,62,63,64,65,66,67,68,69,70,71,72,73,74,75,76,77,78,79,80,81,82,83,84,85,86,87,88,89,90] (see the most relevant studies listed in Table 1 [11,12,13,14,15,16,17,18,19,20,21,22,23,24,25,26], Figure 1—PRISMA flowchart).

The vast majority of patients received re-irradiation with linac-based photons; eight studies reported the use of Cyberknife, six studies reported the use of Gammaknife, and four reported the use of hadron therapy (either with carbon-ions or protons).

The median age at the time of re-irradiation was 54 years (range, 39–69.6 years), and the median first-course RT dose was 60 Gy (range, 54–60 Gy).

### 3.2. Intervention

Re-irradiation was performed with a median time interval of 15.5 months (range, 7–72 months). IDH mutation was present at recurrence in a median proportion of 18% of cases, and MGMT-methylation in 36.35%. Target volume delineation was performed using contrast-enhanced T1-weighted sequences in 60 studies, T2 or FLAIR alone was adopted in eight studies, and combined T1- and T2-weighted sequences were used in 13. Positron emission tomography (PET) imaging was also applied in 13 studies.

The median planning target volume (PTV) size was 33.05 cc (range, 3–205.8 cc); this was generated by applying a median isotropic margin from a clinical target volume (CTV) of 3 mm (range, 1–20 mm). The median total dose for re-irradiation was 30 Gy (range, 20–54 Gy), delivered in a median of five fractions (range, 1–25 fractions). The most frequently applied schedule was 30 Gy in five fractions. Only nine studies reported the simultaneous administration of an integrated boost. Concurrent systemic therapy was administered in a median proportion of 37% of cases (range, 0–100%), mainly consisting of bevacizumab, alone (22 studies) or combined with other systemic agents (temozolomide or pembrolizumab, 5 studies); temozolomide alone was reported in 16 studies and the use of anlotinib (a VEGF-inhibitor) or alisertib (aurora A kinase inhibitor) was reported in one study. In 37 studies, patients received re-irradiation without concurrent systemic therapy.

### 3.3. Outcomes

In the present literature review, re-irradiation was generally well tolerated, with a median incidence of G3 or higher adverse events of 3.5% (range, 0–34.4%).

The median 1-year LC rate after re-irradiation was 61% (range, 18–100%), while the median 1-year PFS rate was 21.7% (range, 7–60%); the median 1- and 2-years OS rates were 43% (range, 18–100%) and 16.7% (range, 5–80%), respectively, with a median overall survival of 11 months (range, 7–29 months) from re-irradiation.

We have also compared the studies according to the preferred MRI sequence in terms of G3 toxicity and clinical outcomes, and interestingly, no statistically significant differences were observed in terms of G3 adverse events (*p* = 0.44), progression-free survival (*p* = 0.49), local control (*p* = 0.86) and overall survival (*p* = 0.70).

Also, the use of PET-based target volume delineation did not influence local control, progression-free survival and overall survival (*p* = 0.17, *p* = 0.97 and *p* = 0.10, respectively).

## 4. Discussion

In this systematic review, our primary objective was to consolidate existing evidence on the most favorable MRI sequence for glioblastoma (GBM) re-irradiation.

Addressing the intricate management of recurrent GBM following initial adjuvant chemo-radiotherapy, the lack of a standardized treatment recommendation underscores the complexity of the therapeutic landscape. Re-irradiation emerges as a viable strategy for recurrent GBM, yet substantial uncertainties persist, encompassing the optimal dose and fractionation, concurrent systemic therapy considerations, and the intricacies of target volume delineation. Navigating these challenges requires a nuanced understanding, and ongoing research endeavors are crucial for refining and individualizing the approach to recurrent GBM, contributing to enhanced patient outcomes.

No standard treatment is currently defined for recurrent glioblastoma. When available, surgical resection can be considered, followed by systemic therapy. Bevacizumab has received FDA approval for its demonstrated benefit in terms of progression-free survival, while no advantage in terms of overall survival has been demonstrated [92,93].

On the contrary, regorafenib showed an overall survival advantage in a randomized phase II trial when compared to lomustine (7.4 months vs. 5.2 months, *p* < 0.001). Alternating electric field therapy showed similar OS outcomes in comparison with chemotherapy in a phase III trial and, to date, low-level evidence is available [94].

In a recent randomized trial led by Tsien et al., the authors explored the potential synergy of re-irradiation (re-RT) with bevacizumab (BEV) in recurrent glioblastoma (GBM) compared to BEV alone. The primary focus was on enhancing overall survival (OS), with secondary considerations for progression-free survival (PFS) and treatment-related adverse events. The trial, NRG Oncology/RTOG1205, featured a prospective phase II design, randomly assigning patients to groups receiving either re-RT (35 Gy in 10 fractions) with concurrent BEV or BEV alone. The results showed no significant OS benefit for BEV + RT but revealed a notable improvement in PFS, particularly at 6 months. The 6-month PFS rate rose from 29.1% for BEV alone, and to 54.3% for BEV + RT. The study concluded that while re-RT was well tolerated and safe, the combination demonstrated a meaningful advancement in PFS without a significant impact on OS in recurrent GBM patients. This marks a significant contribution as the first multi-institutional study evaluating re-RT in recurrent GBM using modern techniques, shedding light on its safety and efficacy [11].

The utilization of radiotherapy for recurrent glioblastoma (GBM) is gaining traction, supported by various instances showing survival rates ranging from 6 to 12 months with limited side effects (5–20%). Despite aggressive initial interventions involving maximal surgical resection and standard external beam radiation therapy (60 Gy/30 fractions) with concurrent and adjuvant temozolomide, almost 90% of WHO grade IV gliomas, particularly GBM, experience local recurrence within 2 years. In the absence of a standardized approach for recurrent GBM, advances in radiation science have introduced reirradiation as a viable strategy. Notably, studies employing stereotactic radiosurgery (SRS) or stereotactic radiotherapy (SRT) in hypofractionated or conventionally fractionated schedules have suggested survival benefits in recurrent GBM patients. However, the effectiveness and potential side effects of a second course of radiation remain unknown. This expanded overview delves into the current state of and recent progress in GBM reirradiation, addressing crucial clinical considerations such as patient selection, radiation techniques, the optimal dose fractionation, brain reirradiation tolerance, and the risk of radiation necrosis.

For the latter topic, a literature review by Minniti et al. reported a range of 0–24% for radiation necrosis; this was especially related to higher RT doses, larger volumes and a reduced risk when the cumulative EQD2 doses were kept below 120 Gy [3].

In the case of larger treatment volumes, several retrospective experiences support the favorable combination of radiotherapy with bevacizumab as a strategy for reducing the risk of radionecrosis, either with external beam radiotherapy or brachytherapy [16].

However, an ongoing debate centers around determining the most effective target delineation strategy that balances enhanced therapeutic outcomes with minimized neuro-toxicity. Intriguingly, our comprehensive review revealed no statistically significant differences in the severe toxicity outcomes across studies, based on the chosen imaging modality (*p* = 0.44). This lack of significance may be attributed to the limited number of studies providing data on this specific outcome. It raises the possibility that the influence of concurrent systemic therapies could contribute to the observed results. Further exploration and analysis, potentially through larger-scale studies, are warranted to gain a more comprehensive understanding of the interplay between imaging modalities, concurrent systemic therapies, and their impact on severe toxicity outcomes in the context of glioblastoma re-irradiation.

In pursuit of this goal, T1-weighted sequences enhanced with contrast medium are widely recognized as the predominant imaging modality for target volume delineation, particularly in the context of stereotactic radiotherapy applications. This consensus aligns with our extensive literature review, encompassing 81 studies, where a majority of 60 studies favor T1-weighted sequences as the preferred imaging modality for re-irradiation. This choice reflects a strategic effort to optimize the clinical benefits and treatment safety. However, challenges persist, as contrast-enhanced T1-weighted imaging may struggle to differentiate between disease progression and the radionecrosis resulting from prior chemo-radiotherapy.

Conversely, there is a hypothesis among some researchers advocating for the inclusion of T2-weighted or FLAIR abnormalities in the clinical target volume (CTV) that these enhance disease control by accounting for the infiltrative component. Notably, our findings underscore the limited adoption of these sequences, with only 8 out of 81 studies incorporating them into their approaches. The scarcity of larger-volume experiences utilizing these sequences is indicative of the ongoing complexities and variations in optimal imaging strategies for glioblastoma re-irradiation.

In our systematic review, we identified 13 studies that advocate for the integration and use of contrast-enhanced (c.e.) T1-weighted and T2/FLAIR sequences as a viable and strategic alternative for enhancing disease control during glioblastoma re-irradiation. This combined imaging approach not only offers a nuanced perspective on tumor characteristics, but also prompts clinicians to explore simultaneous integrated boost (SIB) strategies. The SIB approach involves administering lower doses to the T2/FLAIR-detected areas and higher doses to the c.e. T1 volumes, aiming to achieve a more targeted and effective therapeutic impact. It is noteworthy, however, that despite its potential advantages, this approach remains relatively underexplored in the current literature, with only nine studies identified as embracing this innovative strategy in our review.

The incorporation of amino-acid PET into the toolkit for refining target volume delineation is supported by the research conducted by Miwa et al. in 2014. Their study introduces an innovative treatment approach for recurrent glioblastoma multiforme (GBM) by utilizing hypofractionated stereotactic radiotherapy with intensity-modulated radiation therapy (HS-IMRT), meticulously planned through the fusion of ^11^C-methionine positron emission tomography (MET-PET), computed tomography (CT), and magnetic resonance imaging (MRI). This pioneering method defines the gross tumor volume (GTV) based on the heightened amino acid tracer uptake observed in MET-PET. The resultant treatment, administered at a total dose of 25–35 Gy over 5–7 days, exhibits promising outcomes, including a median overall survival time of 11 months and favorable 6-month and 1-year survival rates. The study’s emphasis on biologic imaging-optimized HS-IMRT lays the groundwork for recognizing the potential use of amino-acid PET in enhancing target volume delineation for recurrent GBM, offering valuable insights beyond the capabilities of conventional MRI alone [20].

Despite lacking prospective validation, several studies highlight the potential of amino-acid PET in distinguishing between pseudoprogression and recurrent disease, with an estimated diagnostic accuracy of around 85%.

The ongoing exploration of innovative modalities like amino-acid PET and novel therapeutic approaches reflects the continuous pursuit of more effective and patient-friendly solutions in managing recurrent glioblastoma. The Phase III trial led by Stupp et al. in 2012 introduced NovoTTF-100A, a portable device delivering electric fields to disrupt cell division. This trial, comparing chemotherapy-free treatment with NovoTTF to active chemotherapy for recurrent glioblastoma, demonstrated mild to moderate TTF-related adverse events, notably a skin rash beneath the transducer arrays. Although the overall survival did improve, the efficacy and activity of this chemotherapy-free device were comparable to conventional chemotherapy regimens for recurrent glioblastoma, with favorable aspects such as reduced toxicity and an enhanced quality of life [95].

Grosu et al. conducted a pioneering study to devise an effective treatment approach for recurrent high-grade gliomas using stereotactic hypofractionated reirradiation guided by biologic imaging, specifically methionine PET. The trial involved 44 patients with various high-grade gliomas, and the treatment planning was based on MET-PET or IMT-SPECT/CT/MRI fusion in 82% of the patients. The study revealed a substantial difference in survival outcomes, with patients treated with PET(SPECT)/CT/MRI imaging experiencing a median survival time of 9 months compared to 5 months for those relying on CT/MRI alone (*p* = 0.03, log-rank). Further analysis demonstrated that patients receiving SFRT based on biologic imaging plus temozolomide had significantly higher median survival times (11 months) compared to those treated without these components (6 months, *p* = 0.008, log rank). The study underscored the feasibility and safety of this multimodal approach, emphasizing the potential impact of biological imaging in treatment planning on survival outcomes, prompting further investigation in larger patient cohorts [49].

In a prospective trial led by Popp et al., the effectiveness of PET-based re-irradiation surpassed that of MRI-based treatments utilizing diffusion-weighted and apparent diffusion coefficient (DWI and ADC) sequences. The study, encompassing 41 patients undergoing fractionated stereotactic re-irradiation for recurrent glioblastoma (rGBM), found that GTV-PET, automatically generated with a tumor-to-background ratio of 1.7–1.8 and manually adjusted, outperformed GTV-ADC_low_; this was manually delineated based on DWI data and ADC maps. Notably, DWI-MRI identified areas with restricted diffusion in 30 of the 41 patients. However, GTV-ADC_low_ exhibited only partial overlap with FET-PET and GdT1w-MRI in rGBM; this prompts further exploration, potentially through histopathological studies, to elucidate the observed imaging disparities [96].

Simultaneously, the ongoing GLIAA, NOA-10, ARO 2013/1 randomized phase II trial, led by Oehlke et al., is investigating the comparative effectiveness of PET-based and MRI-based re-irradiation for GBM. This research stems from the established superiority of amino acid positron emission tomography (AA-PET) in diagnosing gliomas and distinguishing between recurrence and treatment-related changes, contrasting with T1-weighted MRI contrast enhancement. Previous trials demonstrated significant disparities in target volume delineation using AA-PET compared to standard MRI, highlighting its potential impact on survival. The trial protocol outlines a prospective, open-label, randomized, multi-center approach involving 200 patients, utilizing serial MRI scans supplemented by AA-PET scans and/or biopsy/surgery for progression suspicion as primary endpoints. Secondary endpoints include OS, locally controlled survival, volumetric assessment, progression topography, long-term survivors, necrosis localization, quality of life, and safety of FET-application in AA-PET imaging and re-irradiation toxicity. This innovative trial aims not only to improve the outcomes for recurrent GBM patients, but also to establish a standardized methodology for integrating AA-PET and other imaging biomarkers into radiation treatment planning [97].

Nonetheless, PET imaging is expected to be increasingly implemented in the management of recurrent GBM for its potential ability to distinguish the pseudoprogression induced by radionecrosis from high cellularity relapses. [98,99]

Summarizing the collected literature, the conflicting evidence focuses on the need for tailored approaches, in which smaller volumes are to be privileged with the aim of providing a safer treatment. For this purpose, as already recommended in previous studies, c.e. T1-weighted sequences may help clinicians to better identify the target volume for a stereotactic/hypofractionated treatment, with a potentially helpful role in metabolic imaging [100,101,102].

Alongside an improved accuracy of radiotherapy, combination with novel systemic agents might represent a potentially useful therapeutic option, although none of these drugs have demonstrated a clear benefit to date [100].

The present study has several limitations: first of all, most of the literature reported is based on retrospective small cohorts with data frequently missing. Moreover, there is a wide heterogeneity in terms of the radiotherapy approach, including either patients treated with conventional radiotherapy or stereotactic radiotherapy and patients receiving a simultaneous integrated boost. Another inherent bias is linked to the management of a concurrent systemic therapy, a factor that might also have an impact on the toxicity incidence, specifically in this setting. Lastly, the patient selection process is quite heterogeneous among the studies reported and different approaches are collected, with different systemic or local agents often combined with RT, leading to another potential bias in the clinical outcomes assessment. Of note, a comparison between different therapeutic strategies was not the main aim of this literature review.

However, although no statistical differences were observed in terms of clinical outcomes, in the present study, the use of c.e. T1-weighted MRI sequences is the preferred option for glioblastoma re-irradiation. Future studies will explore the possibility of integrating morphologic imaging with functional and metabolic exams, such as spectroscopic MRI or amino-acid PET.

## 5. Conclusions

The evidence gathered in this extensive review strongly supports the prioritization of contrast-enhanced T1-weighted MRI sequences as the preferred imaging modality for glioblastoma re-irradiation. This choice facilitates the administration of higher doses at smaller volumes, either as a standalone approach or in conjunction with systemic therapy. The integration of metabolic imaging emerges as a promising avenue via which to enhance the therapeutic ratio for recurrent glioblastoma re-irradiation. Examining 81 studies encompassing 3280 patients, this review provides insights into re-irradiation practices, highlighting variations in techniques such as linac-based photons, Cyberknife, and Gammaknife. Despite these differences, no statistically significant variations were observed in G3 adverse events, progression-free survival, local control, and overall survival based on the preferred MRI sequence or the use of PET-based target volume delineation. The review underscores the ongoing challenges in establishing a standard treatment for recurrent glioblastoma, emphasizing the need for personalized and innovative approaches to improve clinical outcomes in this formidable clinical scenario.

## Figures and Tables

**Table 1:** Summary of the most relevant experiences of re-irradiation for glioblastoma.

Author (Year)	Patients (n)	Median Age	Median Time to Re-RT	Median PTV	MRI-Sequence	Use of PET	Concurrent Systemic Therapy	Radiotherapy	G ≥ 3Toxicity	Clinical Outcomes
Ciammella (2022) [12]	12	60.5	11	45.7 (15–97.1)	c.e.T1-weighted plus ADC	no	no	30–50 Gy/5 fx	8.3%	1-yr OS = 42%
Navarria (2022) [13]	90	54	24	98.9 (1.7–505)	c.e.T1-weighted	Yes (11CMET-PET)	53.3%	25 Gy/1 fx; 37.5 Gy/5 fx; 49.5 Gy/15 fx	10%	1-yr OS = 66.7%
Breen (2021) [13]	20	53	21.5	NA	c.e.T1-weighted	Yes (DOPA-PET)	no	35 Gy/10 fx	20%	1-yr OS = 75%
Sahebjam (2020) [14]	32	55.5	NA	55	c.e.T1-weighted	no	100% (bevacizumab + pembrolizumab)	30 Gy/5 fx	34.4%	1-yr LC = 83%; 1-yr OS = 58%
Cohen (2020) [24]	22	55.5	NA	192.3	c.e.T1-weighted	no	100% (bevacizumab + mynocicline)	37.5 Gy/15 fx	24%	6.4 mo OS
Fleischmann (2019) [16]	161	51	18	118	c.e.T1-weighted	no	77%(bevacizumab)	36 Gy/13 fx	10%	9 mo OS
Song (2019) [17]	17	61	12	34.2	c.e.T1-weighted + T2	no	100% (alisertib)	35 Gy/10 fx	3.3%	1-yr PFS = 10%; 1-yr OS = 50%
Moller (2017) [18]	31	54	23	67	c.e.T1-weighted	no	no	35 Gy/10 fx	0%	7 mo OS
Clarke (2017) [19]	15	63	NA	NA	c.e.T1-weighted (T2/FLAIR at phyisician’s discretion)	no	100% (bevacizumab)	33 Gy/3 fx	6.7%	1-yr PFS = 20%; 1-yr OS = 51%
Miwa (2014) [20]	21	53.9	12	27	c.e.T1-weighted	Yes (11CMET-PET)	no	30 Gy/5 fx	0%	1-yr PFS = 14.5%; 1-yr OS = 38.1%
Greenspoon (2014) [25]	31	53	NA	12.1	c.e.T1-weighted	no	100% (temozolomide)	25–35 Gy/3 fx	4%	9 mo OS
Cabrera (2013) [26]	15	53	20	NA	T2/FLAIR	no	100% (bevacizumab)	25 Gy/5 fx	1%	14.4 mo OS
Minniti (2013) [23]	54	52	15.5	30.3	c.e.T1-weighted	Yes (18FDOPA-PET)	100% (temozolomide)	30 Gy/6 fx	21%	1-yr PFS = 24%; 1-yr OS = 53%
Minniti (2011) [22]	36	56	14	32.1	T2/FLAIR	no	100% (temozolomide)	37.5 Gy/15 fx	1%	1-yr PFS = 8%; 1-yr OS = 33%
Vordermark (2005) [21]	19	50	19	15	T2/FLAIR	no	100% (nimustine + teniposide + temozolomide)	NA	0%	1-yr OS = 26%

## Data Availability

Data are available upon request.
